# The Texas Medical Law Upheld

**Published:** 1912-03

**Authors:** 


The Texas Medical Law Upheld.—One Ira Collins, an Osteopath at El Paso, Texas, was convicted by the local court of violation of the law, in that he was practicing medicine without a license from the State Board of Medical Examiners. He appealed to the Supreme Court of the United States, contending that the law is unconstitutional; that he had a diploma from an Osteopathic College, and therefore he had a right to practice Osteopathy; and that as he gave no medicine he was not practicing medicine. On February 19th (ult.) the United States Supreme Court held that he was practicing medicine within the meaning of the law, and upheld the decision of the lower court.

That settles it for all time. Attorney General Lightfoot argued the case at Washington. This Collins, it will be remembered, was appointed by Governor Campbell as the Osteopathic member and representative of that "cult" on the State Board of Medical Examiners (mixed), and he made a handle of the position to boost himself in the newspapers. Campbell then canceled the appointment, and Collins refused to be examined.

